# A fixed brain seeded amplification assay to complement neuropathological prion disease diagnosis

**DOI:** 10.1093/jnen/nlaf105

**Published:** 2025-09-03

**Authors:** Victoria Lewis, Laura Ellett, Enie Lei, Christiane Stehmann, Ian Birchall, Matteo Senesi, Catriona McLean, Steven J Collins

**Affiliations:** Department of Medicine (RMH), The University of Melbourne, Parkville, VIC, Australia; Australian National Creutzfeldt-Jakob Disease Registry (ANCJDR), The Florey Institute of Neuroscience and Mental Health, Parkville, VIC, Australia; The Florey Institute of Neuroscience and Mental Health, Parkville, VIC, Australia; The Florey Institute of Neuroscience and Mental Health, Parkville, VIC, Australia; Australian National Creutzfeldt-Jakob Disease Registry (ANCJDR), The Florey Institute of Neuroscience and Mental Health, Parkville, VIC, Australia; The Florey Institute of Neuroscience and Mental Health, Parkville, VIC, Australia; The Florey Institute of Neuroscience and Mental Health, Parkville, VIC, Australia; Department of Medicine (RMH), The University of Melbourne, Parkville, VIC, Australia; Australian National Creutzfeldt-Jakob Disease Registry (ANCJDR), The Florey Institute of Neuroscience and Mental Health, Parkville, VIC, Australia; The Florey Institute of Neuroscience and Mental Health, Parkville, VIC, Australia; Department of Anatomical Pathology, Alfred Health, Melbourne, VIC, Australia; Department of Medicine (RMH), The University of Melbourne, Parkville, VIC, Australia; Australian National Creutzfeldt-Jakob Disease Registry (ANCJDR), The Florey Institute of Neuroscience and Mental Health, Parkville, VIC, Australia; The Florey Institute of Neuroscience and Mental Health, Parkville, VIC, Australia

**Keywords:** formalin-fixed paraffin-embedded, prion, prion seeding, PrP, PrP^Sc^, RT-QuIC

## Abstract

Prion diseases are rare neurodegenerative disorders that share misfolding of the normal cellular prion protein into disease-causing isoforms known as “prions” as the critical pathophysiological event. Definite diagnosis can only be achieved through neuropathological confirmation. The neuropathological features of prion disease are well described; however, some molecular subtypes are typified by characteristic neuropathological features that are subtle or absent. Prion seeding assays have excellent specificity and have considerably improved premortem diagnostic accuracy but they have reduced sensitivity for some uncommon prion disease molecular subtypes. We developed a formalin-fixed, paraffin-embedded tissue-based prion seeding assay to serve as a complementary diagnostic tool for prion diseases. Fixed brain tissue was prepared through an optimized process involving careful defacing of tissue blocks prior to sampling and then stepwise deparaffinization and homogenization. Fixed tissue homogenates are then tested in an adapted version of a diagnostic cerebrospinal fluid (CSF) prion seeding assay, which utilizes full-length recombinant hamster prion protein as substrate. Two examples illustrate the utility of the assay by confirming prion seeding in fixed brain tissue from previously neuropathologically misdiagnosed obligate carriers of 2 different prion protein gene mutations. The importance of careful tissue sampling to rigorously maintain the diagnostic specificity of this assay is also highlighted.

## INTRODUCTION

Prion diseases comprise a group of rare and typically rapidly progressive neurodegenerative diseases with protean clinical features that often overlap with those of more common neurological illnesses.^1^ As a result, a definite diagnosis of prion disease can only be achieved through neuropathological examination, most often performed postmortem. Neuropathological features typical of prion disease include vacuolation of the neuropil, neuronal loss, reactive astrocytic gliosis, and the deposition of disease-associated isoforms of the prion protein (PrP^Sc^) detected either immunohistochemically or biochemically.^2^

The development of disease-specific PrP^Sc^ seeding assays, such as “real-time quaking induced conversion” or “RT-QuIC”, especially when applied to CSF, has provided a great advancement in the premortem diagnosis of prion disease. The primary cause of human prion disease is through the aberrant misfolding of the normal cellular prion protein, PrP^C^, into a pathogenic isoform, PrP^Sc^. Cumulative evidence strongly indicates that different stable misfolded conformations of PrP^Sc^ exist, thought they are to be the basis of different prion “strains,” which correlate with different clinical profiles and molecular subtypes.^3^^,^^4^ Critical to prion disease pathogenesis is the ability of PrP^Sc^ to recruit PrP^C^ and through an incompletely understood templating mechanism involving binding to PrP^C^ by which PrP^C^ is induced to misfold and convert into PrP^Sc^, thereby progressively leading to a toxic accumulation of PrP^Sc^, neurodegeneration and death.^5^ The RT-QuIC assay exploits this “templated conversion” property of PrP^Sc^ and amplifies very low levels of PrP^Sc^ seed present in biological samples.^6^^,^^7^ While RT-QuIC using patient CSF is near 100% disease-specific, its sensitivity is somewhat lower (typically between 80% and 90%), with certain prion disease strains such as the rarer molecular subtypes MM2C and MM2T (also called sporadic fatal insomnia or “sFI”) as well as variably protease sensitive prionopathy (VPSPr) far less likely to test positive, especially in first generation assays.^8–10^ The reasons for reduced sensitivity of the RT-QuIC in some prion disease subtypes is not well understood but it appears to depend on a combination of factors, including assay parameters such as the recombinant prion protein substrate or shaking speed and/or shake/pause pattern, prion disease molecular subtype, and the time of sampling relative to disease progression. Importantly, despite this prion-specific biomarker test, neuropathological confirmation of prion disease remains the “gold standard,” and a requirement for a “definite” case classification.

Typical neuropathological features of prion disease are well known but some prion disease cases may display subtle or very modest neuropathological changes, including the absence of immunohistochemically detectable disease-associated PrP^Sc^.^2^ Most often this occurs in the rarer molecular subtypes, such as MM2T/sFI, which may have severe neuronal loss in the thalamus, but other pathological features including characteristic spongiform change are mild or absent throughout the brain; moreover, PrP^Sc^ deposition is patchy, restricted to specific regions and is sometimes undetectable. Similarly, VPSPr has unique histological and immunohistochemical patterns, with varied intensity of detectable PrP^Sc^ deposition, also dependent on the specific anti-PrP antibody employed. Supplementary laboratory-based biochemical tests for the detection of PrP^Sc^ in fresh/frozen brain tissue can be used to complement neuropathological analysis and confirm prion disease diagnosis. Notably this includes the use of sodium phosphotungstic acid (NaPTA) treatment to selectively precipitate^11^ and concentrate PrP^Sc^ from much larger volumes of brain than would typically be possible to analyze thereby increasing detection sensitivity; however, if there is no frozen tissue available, this additional highly sensitive NaPTA testing is not possible.

Herein, we describe the development and utility of a fixed brain tissue RT-QuIC (FxBRTQ) assay that the Australian National Creutzfeldt-Jakob Disease Registry (ANCJDR) employed to aid in the diagnosis and classification of 2 previously misdiagnosed cases of genetic prion disease.

## METHODS

### Ethics statement

The ANCJDR has human research ethics approval for the use of retained diagnostic tissue for assay development (University of Melbourne Human Research Ethics Committee approval# 2023-20362-37091-3). Written informed consent for participation was not required for this study in accordance with the nation legislation and institutional requirements.

### Tissue sampling

Formalin-fixed tissue was sampled from paraffin-embedded blocks from 4 confirmed prion disease cases including 1 where blocks were not formic acid treated (CJD1), and 3 where blocks had been treated in formic acid (CJD2-4). There were 6 confirmed “nonprion disease” controls. These included 3 that were at 1 point “suspected” CJD cases and had been processed and sampled using the dedicated prion histology equipment (C1-3), and 3 that had never entered the dedicated prion diagnostic histology laboratory (C4-6). Using a microtome, 9-10-µm tissue “curls” were taken. Importantly, after the potential of cross-contamination had been detected, careful defacing of blocks and the use of clean/new microtome blades at each step of defacing and sampling was employed. For one of the cases studied, the only available tissue was fixed brain tissue mounted on a single unstained slide; sampling involved carefully using a scalpel to scrape this tissue from the slide into a microcentrifuge tube prior to deparaffinization.

### Deparaffinization and homogenization

**Figure 1.**
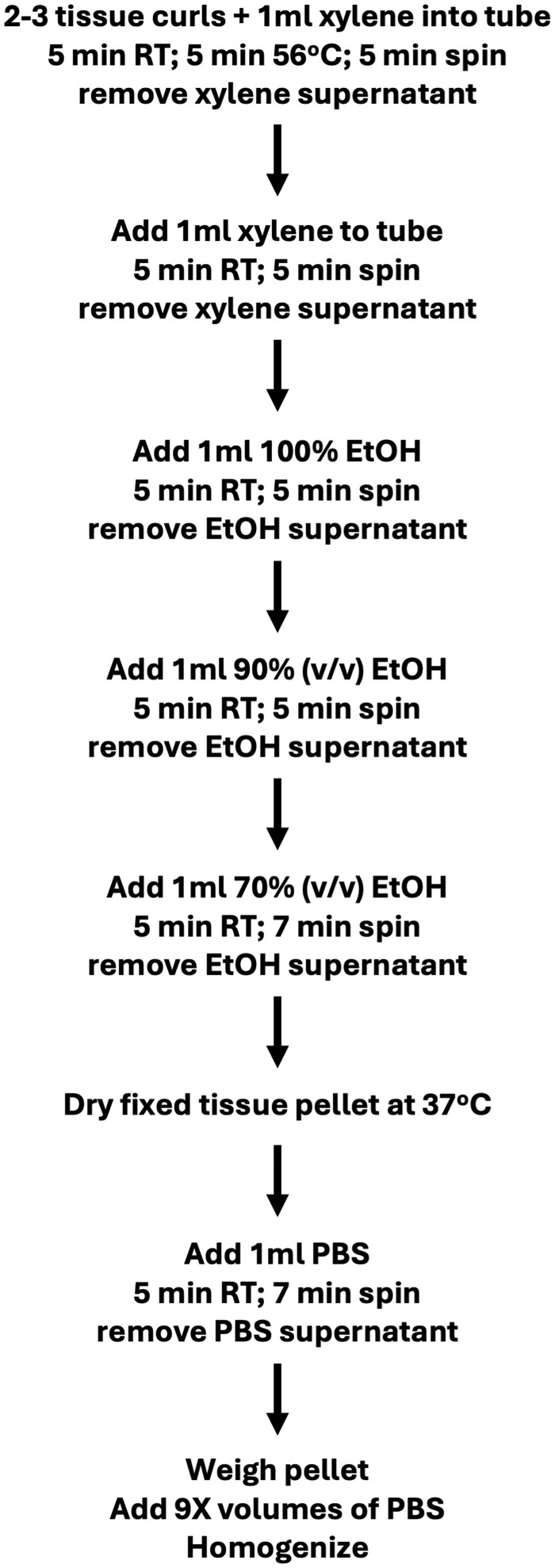
Flow diagram of deparaffinization protocol. Room temperature (RT) incubations were with gentle agitation using an ELMI Intelli-Mixer set at “U2 95.” The 56 ºC incubation was with gentle agitation in an Eppendorf Thermomixer set at 300 rpm. All centrifugations were at 16 900*g*, RT, for the time (minutes) as indicated. Ethanol (EtOH) was diluted with MilliQ water to the final percent volume/volume (v/v) as indicated.

Deparaffinization of tissue curls was adapted from Hepker et al who developed an assay for testing formalin-fixed paraffin-embedded (FFPE) tissue in an α-synuclein seeding assay.^12^ Briefly, and as depicted in Figure 1, 2-3 FFPE tissue curls were added to a preweighed screw-capped microcentrifuge tube, 1 mL xylene was added and tube was incubated for 5 min at room temperature with agitation, followed by 5 min at 56 ºC with agitation, 5 min of centrifugation at 16 900*g*, and careful removal of the xylene supernatant. A second xylene treatment was performed, identical to the first but omitting the 56 ºC incubation. Stepwise ethanol treatments (100%, 95%, 70%) were then carried out identical to the second xylene treatment, with the final centrifugation after 70% ethanol incubation increased to 7 min. The fixed tissue pellet was dried at 37 ºC to evaporate any residual ethanol, then the pellet was “washed” with 1 mL of phosphate buffered saline (PBS) following the same incubation/centrifugation as the 70% ethanol. The washed pellet was weighed and a 10% w/v PBS fixed tissue homogenate was made using a BeadBug homogenizer and ceramic beads. Homogenates were stored at −80 ºC.

### Fixed brain RT-QuIC assay

The fixed brain RT-QuIC assay (FxBRTQ) is a modified version of the ANCJDR’s diagnostic CSF RT-QuIC assay, which uses recombinant full-length hamster PrP (recPrP) produced in-house as the substrate.^6^^,^^9^^,^^13^ Briefly, 100 µL of reaction buffer was loaded per well, containing a final concentration of 10 mM PBS (pH 7.4), 170 mM NaCl (total 400 mM including the PBS), 10 µM Thioflavin T, 10 µM EDTA, 0.1 mg/mL recPrP and 2 µL of the indicated homogenate (% w/v) mixed with 13 µL artificial CSF (aCSF; 125 mM NaCl, 2.5 mM KCl, 1 mM MgCl_2_, 2 mM CaCl_2_, 25 mM HEPES, 500 mg/L Glucose, 200 mg/L BSA). During assay development, frozen positive (PM proven CJD; FrzPOS) and negative (PM proven not-CJD; FrzNEG) brain were included as additional controls. Technical controls included aCSF in place of brain homogenate (aCSF), and aCSF with recPrP omitted from the reaction mixture (aCSF-PrP). Samples were tested in 4 replicate wells per independent assay. Assays were run on a BMG Fluostar Omega, with cycles of 900 rpm, shaking of 84 s and rest of 36 s.

## RESULTS

### FxBRTQ assay development

**Figure 2.**
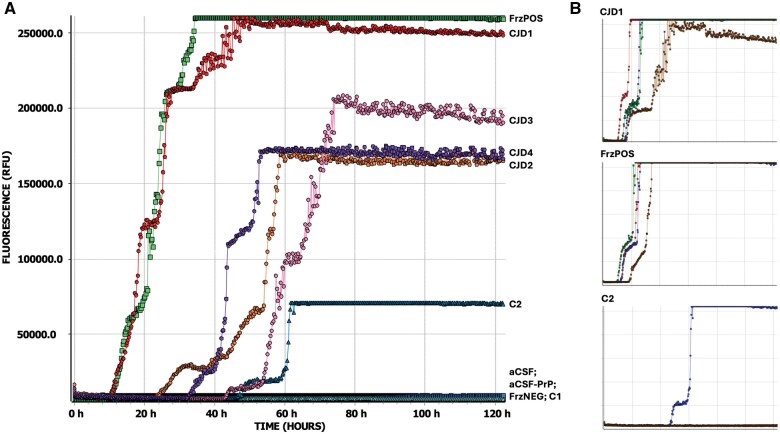
Fixed brain RT-QuIC assay development. (A) Average curves for each 1% (w/v) homogenate tested, including fixed CJD brain (CJD1, CJD2, CJD3, CJD4), frozen CJD brain (FrzPOS), fixed control (non-CJD) brain (C1, C2), frozen control (non-CJD) brain (FrzNEG). All CJD samples were positive in 4/4 wells; C2 was positive in 1/4 wells. Technical controls were aCSF with (aCSF) or without (aCSF-PrP) recombinant PrP in the reaction mixture. (B) Single well results of the averages presented in (A) for nonformic acid-treated fixed CJD brain (CJD1), frozen CJD brain (FrzPOS), and the false-positive fixed negative control brain (C2). For all graphs: *y*-axis is ThT fluorescence measurement (relative fluorescence units—RFU), with grid lines at 50 000 RFU and the maximum value detectable in plate reader 260 000 RFU; *x*-axis is time of assay (hours), with grid lines every 20 h.

**Figure 3.**
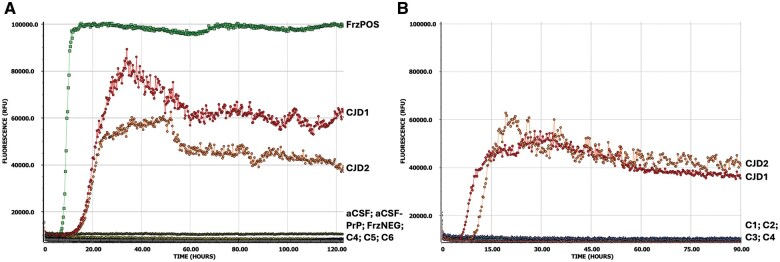
Careful fixed tissue sampling abolishes false-positive RT-QuIC results in non-CJD controls. (A) Average curves for each 1% (w/v) homogenate tested, including fixed CJD brain (CJD1, CJD2), frozen CJD brain (FrzPOS), fixed control (non-CJD) brains which were sampled away from the prion histology laboratory (C4, C5, C6), frozen control (non-CJD) brain (FrzNEG). Technical controls were aCSF with (aCSF) or without (aCSF-PrP) recombinant PrP in the reaction mixture. (B) Average curves for each 1% (w/v) homogenate tested, including fixed CJD brain (CJD1, CJD2) and fixed control (non-CJD) brains sampled away from the prion histology laboratory (C4) or original control brains carefully resampled (C1, C2) and additional new control (C3) sampled after defacing and with new microtome blades in the prion histology laboratory. For all graphs: all CJD samples were positive in 4/4 wells; *Y*-axis is ThT fluorescence measurement (relative fluorescence units—RFU), with grid lines at 20 000 RFU; *X*-axis is time of assay (hours), with grid lines every 20 h (A) or 15 h (B).

Our initial attempts at testing 1% (w/v) fixed brain homogenate, utilizing the existing CSF diagnostic RT-QuIC assay and replacing CSF with diluted brain, found that fixed CJD samples were able to efficiently seed recombinant full-length hamster prion protein misfolding as indicated by the rapid and high amplitude increase in ThT fluorescence in all 4 wells from each of the 4 CJD cases (Figure 2A). Notably the CJD1 nonformic acid-treated fixed brain gave RT-QuIC responses almost identical to those of the frozen CJD brain control (FrzPOS) (Figure 2B). One false positive was observed, with PrP seeding seen in 1 of 4 wells from 1 fixed control (C2) (Figure 2A and B). The assay was repeated, making new fixed tissue homogenate on this individual and including frozen brain tissue homogenate from the same patient, with the fixed control samples again testing false positive (Figure S1); however, the frozen specimen tested negative, confirming the individual as non-CJD and indicating a potential issue with the fixed tissue sampling. One concern was whether the fixation process itself may have imparted some seeding capacity onto PrP^C^; therefore, new control tissue curls were sampled from cases that had never been suspected as CJD and were never housed or processed in the prion histology laboratory (C4-C6). When C4-C6 were tested, there was no evidence of PrP seeding (Figure 3A). To confirm whether the false-positive results were therefore due to cross-contamination inherent in processing and use of the dedicated prion histology equipment, the 2 original control cases (C1 and C2), and an additional control sample (C3) were carefully resampled, using the prion equipment but new microtome blades at each step of defacing the blocks and sampling the tissue curls for deparaffinization; further FxBRTQ testing showed no evidence of PrP seeding from these control samples (Figure 3B).

### Application of the FxBRTQ assay at the ANCJDR

#### Case #1

**Figure 4.**
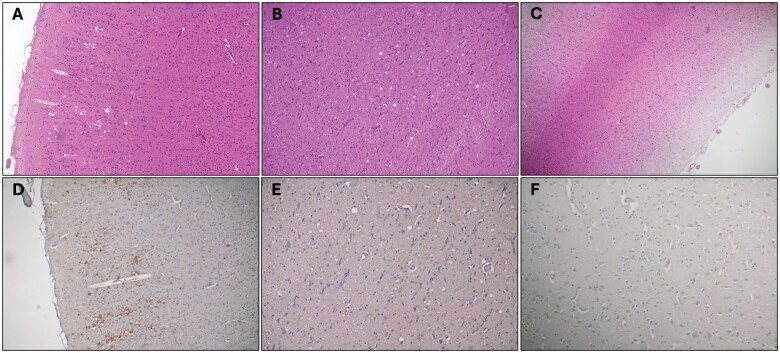
Illustration of subtle or absent neuropathological features of prion disease. Representative images from Case #1 occipital cortex (A, D) and thalamus (B, E) depicting focal spongiform encephalopathy, neuronal loss, and gliosis with 12F10 PrP immunoreactivity; and Case #2 frontal cortex (C, F) showing atrophy and spongy change to lamina 2 with gliosis and neuronal loss with no evidence of a spongiform encephalopathy and no evidence of PrP immunoreactivity. Hematoxylin and eosin-stained slide images in (A) and (B) at 100× magnification and (C) at 40× magnification. Anti-PrP 12F10 antibody-stained slide images in (D) and (E) at 100× magnification and (F) at 200× magnification.

**Figure 5.**
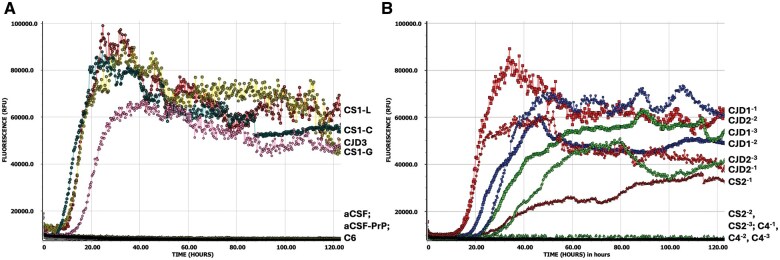
Application of the FxBRTQ by the ANCJDR confirms prion seeding in misdiagnosed inherited prion disease cases. (A) Average curves for each 1% (w/v) homogenate tested, including fixed positive control CJD brain (CJD3), fixed negative control non-CJD brain (C6), and 3 brain regions from Case #1 (CS1) from blocks “C,” “G,” and “L.” All CJD and CS1 samples were positive in 4/4 wells. Technical controls were aCSF with (aCSF) or without (aCSF-PrP) recombinant PrP in the reaction mixture. (B) Average curves for each sample 1% (^−1^), 0.1% (^−2^), and 0.01% (^−3^) w/v homogenate tested, including fixed CJD brain (CJD1, CJD2), fixed non-CJD control brain (C4) and Case #2 (CS2). All CJD samples were positive in 4/4 wells; CS2^−1^ was positive in 3/4 wells. For all graphs: *Y*-axis is ThT fluorescence measurement (relative fluorescence units—RFU), with grid lines at 20 000 RFU; *X*-axis is time of assay (hours), with grid lines every 20 h.

The ANCJDR received a CSF sample from a patient for diagnostic biomarker screening, which subsequently tested positive in the RT-QuIC assay, as well as for both nonspecific biomarker proteins, 14-3-3 and total-Tau. A limited postmortem revealed typical CJD neuropathology, which was not unexpected given the CSF RT-QuIC result. As part of routine surveillance, and to confirm disease etiology, the ANCJDR seeks relevant medical, family, and social history information on confirmed CJD cases. We uncovered that approximately 20 years prior the ANCJDR had been involved in the investigation of a second degree relative, designated hereafter as “Case #1,” notified to the ANCJDR at that time for CSF biomarker analysis; however, the CSF was hemorrhagic and unsuitable for testing. Case #1 had been referred for a brain postmortem, and after neuropathological evaluation including immunohistochemistry with the anti-PrP antibody 3F4, CJD was ruled out. The diagnoses made were (1) incidental pedunculated subependymoma in the fourth ventricle, (2) small amount of diffuse white matter edema, and (3) one small area of patchy superficial vacuolation in the occipital cortex (considered probably agonal in nature) with no clear evidence of CJD; the case was removed from the ANCJDR register. Nonetheless, the clear-cut CJD diagnosis in the more recent second degree relative prompted a review of the Case #1 brain tissue. Thorough neuropathological review and immunohistochemical staining with a different anti-PrP antibody (12F10) uncovered a single occipital cortical focus and a few thalamic foci with some “typical” neuropathological features of prion disease (vacuolation, gliosis, cortical neuronal loss) including PrP immunoreactivity (Figure 4A, B, D and E). When considering these 2 anti-PrP antibodies, despite both being directed toward the prion protein mid-region with the epitope for 3F4 approximately 30 residues closer to the flexible N-terminus, only 12F10 may show selective labeling of disease-associated PrP.^14^ What is clear is that slightly different antibody epitopes give rise to the detection of different morphological PrP deposits and staining patterns, also dependent on prion strain or molecular subtype being analyzed,^14^ which may provide the explanation for the difference in PrP detection seen in Case #1. The neuropathological diagnosis of Case #1 was revised to CJD noting, “this is an unusual pattern of distribution with only 1 focus in the cerebral cortex and a predominantly thalamic involvement of spongiform change, astrocytic gliosis, and 12F10 immunoreaction.” For supplementary confirmation of prion disease, we tested fixed tissue from the 2 regions of focal neuropathological changes (occipital cerebral cortex and thalamus), as well as an “unremarkable” region (cerebellum), and found strong evidence of prion seeding, with rapid conversion in all wells of all 3 regions tested (Figure 5A). A family history of neurological disease involving older generations was subsequently revealed, and analysis of the prion protein gene (*PRNP)* in frozen brain tissue-extracted DNA detected a pathogenic E200K mutation in the recently confirmed CJD relative. Given the obligate carrier status of Case #1, they were ultimately reclassified as genetic prion disease.

#### Case #2

The ANCJDR was notified of an alive symptomatic case of inherited prion disease with a confirmed pathogenic *PRNP* 6-octapeptide repeat insertion mutation detected. This individual had a strong family history of a neurogenerative disease, (which had been previously diagnosed as frontotemporal lobar degeneration-U [FTLD-U]), and no other diagnostic tests for prion disease (ie, CSF biomarkers or brain MRI) had been performed. The ANCJDR established that a first degree relative, hereafter designated “Case #2,” had been investigated decades earlier (1995) for potential prion disease to explain a very long duration of a neurodegenerative illness, but was diagnosed as “frontal lobe degeneration of non-Alzheimer type” following postmortem brain examination. This original neuropathology report noted severe atrophy of the left and right superior frontal cortices, with neuronal loss most associated with reactive gliosis and prominent status spongiosis affecting the second and third cortical layers with no Pick bodies, Lewy bodies, or ballooned neurons. The temporal, parietal, and occipital cortices were spared. Several years later the ANCJDR had facilitated a neuropathological review of available blocks from the superior frontal cortex and hippocampus from Case #2, including antiprion protein immunostaining with 12F10 which had originally not been performed (Figure 4C and F), with no immunoreaction evident. Additional immunoperoxidase staining revealed very occasional neurons immunoreactive with p62 in the cell cytoplasm with Tau and TDP43 showing no immunoreaction. The conclusion aligned to that of the original neuropathology report and thought to best fit a diagnosis of “frontotemporal dementia of no specific subtype.” Upon gaining knowledge of the alive symptomatic first degree relative, we tested the only retrievable fixed tissue from Case #2: a single mounted paraffin-embedded section from the superior frontal cortex. The tissue was removed from the slide, deparaffinized, homogenized, and tested in the FxBRTQ, with clear evidence of PrP seeding seen (Figure 5B). Given the obligate carrier status of Case #2, they were ultimately reclassified as genetic prion disease.

## DISCUSSION

The RT-QuIC assay is a highly specific investigative tool for premortem diagnosis of prion disease when applied to biofluids such as CSF. Herein, we propose the use of FxBRTQ as an auxiliary diagnostic test to neuropathological interrogation in cases of unresolved, atypical, or complicated potential prion disease. Neuropathologists are sometimes presented with cases in which anti-PrP immunostaining is negative or equivocal despite other typical features being present, where neuropathological changes are very modest (such as highly focal), or where antibody specificity or artifact is in question. Traditionally, in these more complicated situations, detection of PrP^Sc^ by western blotting, often utilizing NaPTA precipitation of PrP^Sc^ to increase the sensitivity of detection, is invoked. Unfortunately, fresh frozen brain tissue may not be available for these analyses for a variety of reasons, potentially associated with specimen storage and/or infection control concerns in the mortuary or laboratory, or for historical or archival cases requiring periodic review and where fresh tissue was not taken or kept indefinitely. In such instances, our study has shown the utility of analyzing fixed brain tissue using RT-QuIC.

Of the definite CJD positive controls utilized for our assay development, only 1 has undergone molecular subtyping (CJD3 is a T2MM, as per the “London/Collinge nomenclature”^15^) Nevertheless, CJD2 and CJD4 were both known to be *PRNP* polymorphic codon 129 (c129) valine homozygotes. Whether all other molecular subtypes of human prion disease, especially the rarer forms wherein neuropathological confirmation may be more challenging such as sFI, fatal familial insomnia, or VPSPr, are able to convert in the FxBRTQ remains to be determined, though we and others have found these prion disease molecular subtypes, as well as some of the more common sporadic and genetic subtypes, readily convert using frozen brain specimens (Figure S2).^16^ Although no direct comparisons were made testing fixed vs frozen tissue from the same individual, given the high sensitivity of the FxBRTQ it is reasonable to assume FFPE tissue preserved from at least all molecular subtypes of human prion disease that have previously tested positive with frozen brain would also have detectable seeding.

Dong et al recently showed high capacity for prion seeding in the RT-QuIC using fixed tissue that was not paraffin embedded from all the major sporadic CJD molecular subtypes associated with c129 methionine (M) homozygosity, in an assay which utilized full-length recombinant human c129M prion protein.^17^ Concordant with our observation, Dong et al found significant but reduced seeding in samples that had been treated with formic acid, which is typically utilized to inactivate prion infectivity and for antigen retrieval. Their analysis of the RT-QuIC as an end-point assay allowed for quantification of a 3-log reduction in seeding after formic acid treatment compared to formalin fixation alone, the former treatment having previously been shown to significantly reduce prion infectivity by 5- to 6-logs such that for some animal prion strains at least, there is no disease transmission with bioassay.^18^ Additionally, Dong et al determined that the time in storage for the fixed tissue decreased seeding, which may account for the lower amplitude and longer lag seen in our Case #2, where the sample recovered for the FxBRTQ was over approximately 30 years old. The ultrasensitive and adaptable nature of the RT-QuIC may lend itself to diverse applications including for prion strain typing, surface detection of prions for workplace safety, and assessment of efficacy of sterilization methods for example of medical/surgical equipment, among others.^19–21^

Rigorously maintaining diagnostic specificity when employing a highly sensitive technique is critical to avoid potential “over-diagnosis,” including for prion disease. Indeed, Nakagaki et al recently detected seeding in frozen brain from an asymptomatic cadaver despite neuropathological examination finding no spongiform change or PrP^Sc^ deposition, suggesting they may have uncovered a preclinical case of prion disease.^22^ Meticulously harvesting brain seeding samples and strict incorporation of appropriate controls for FxBRTQ are paramount to ensure high specificity in clinically symptomatic cases with neuropathological uncertainty, in order to rule in/out a prion disease diagnosis. Such an approach is analogous to the way the ANCJDR undertakes biochemical testing of frozen brain in a standardized protocol with the appropriate positive and negative controls to provide a “PrP^Sc^ detected” or “PrP^Sc^ not detected” outcome summary. Illustrating that rigorous validation is required before techniques such as FxBRTQ can be routinely implemented as a standard or recommended laboratory test for prion diagnosis, a similar fixed tissue assay has very recently been reported in a larger series of cases.^23^ Excellent specificity and sensitivity were achieved in this study by careful calibration of the assay to maximize dichotomizing cut points and rechallenging of results that were not straightforward or consistent with expected outcomes. For prion disease diagnostic centers, especially those already housing the infrastructure for diagnostic antemortem RT-QuIC assays but where handling fresh/frozen brain tissue might be prohibited or a challenge, this novel approach, including after formic acid treatment of brain blocks, has the potential to improve surveillance and reporting of prion diseases.

## Supplementary Material

nlaf105_Supplementary_Data
