# Supplementary material for: A fixed brain seeded amplification assay to complement neuropathological prion disease diagnosis
Source: J Neuropathol Exp Neurol. 2025 Sep 3;85(1):17–23. doi: 10.1093/jnen/nlaf105 (PMC12744881; doi:10.1093/jnen/nlaf105)
Supplement: nlaf105_Supplementary_Data [file nlaf105_supplementary_data.zip › LEWIS-Supp Figs.pdf]

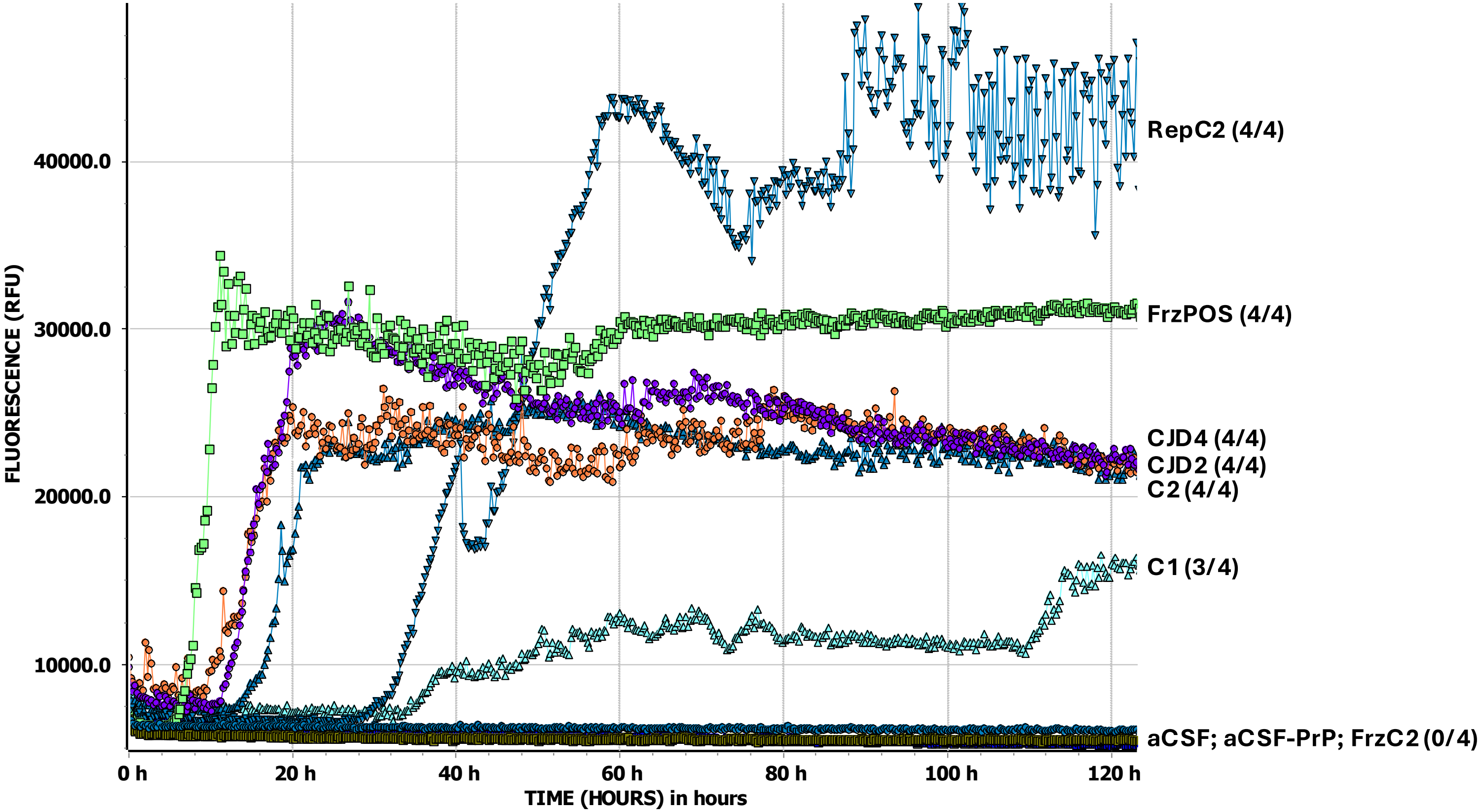

Supp Fig. 1. Fixed brain RT-QuIC assay development. Average curves (wells positive / four wells) for each 1% w/v homogenate tested, including fixed CJD brain (CJD2, CJD4), frozen CJD brain (FrzPOS), fixed control (non-CJD) brain (C1, C2), a repeat sampling of C2 (RepC2) and frozen brain from the same C2 individual (FrzC2). Technical controls were aCSF with (aCSF) or without (aCSF-PrP) recombinant PrP in the reaction mixture. For all graphs: Y-axis is ThT fluorescence measurement (RFU), with grid lines at 10,000 RFU; X-axis is time of assay (hours), with grid lines every 20 hours.

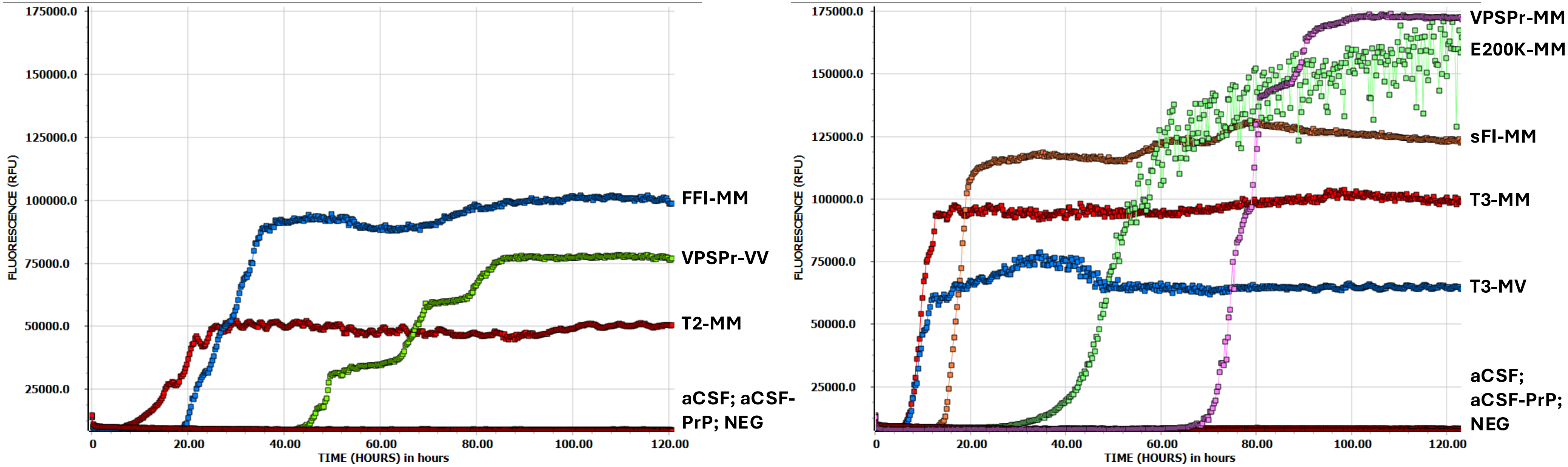

Supp Fig. 2. Frozen brain based RT-QuIC assays show seeding in a range of human prion disease subtypes. Representative average curves for each 1% w/v frozen brain homogenate of various prion disease subtypes tested (as per labelled curves), and negative non-CJD control (NEG). Technical controls were aCSF with (aCSF) or without (aCSF-PrP) recombinant PrP in the reaction mixture. For all graphs: All prion disease subtypes tested were positive in 4/4 wells; Y-axis is ThT fluorescence measurement (RFU), with grid lines at 25000 RFU; X-axis is time of assay (hours), with grid lines every 20 hours.
